# Average daily gain divergence in beef steers is associated with altered plasma metabolome and whole blood immune-related gene expression

**DOI:** 10.1093/tas/txaa074

**Published:** 2020-05-27

**Authors:** Ibukun M Ogunade, Megan McCoun

**Affiliations:** College of Agriculture, Communities, and the Environment, Kentucky State University, Frankfort, KY

**Keywords:** average daily gain, beef steers, metabolomics, plasma

## Abstract

We evaluated the plasma amine/phenol- and carbonyl-metabolome and whole-blood immune gene expression profiles in beef steers with divergent average daily gain (ADG). Forty-eight Angus crossbred beef steers (21 days postweaning; 210 ± 8.5 kg of body weight) were fed the same total mixed ration ad libitum for 42 days with free access to water. After 42 days of feeding, the steers were divided into two groups of lowest (LF: *n* = 8) and highest ADG (HF: *n* = 8). Blood samples were taken from all steers. The blood samples from LF and HF steers were used for further analysis. A subsample of the whole blood was immediately transferred into RNA-protect tubes for RNA extraction and messenger RNA expressions of 84 genes involved in innate and adaptive immune responses. Another subsample of the whole blood was immediately centrifuged to harvest the plasma for subsequent metabolome analysis. The average daily dry matter intake of the steers in LF and HF was 6.08 kg ± 0.57 and 6.04 kg ± 0.42, respectively, and was similar between the two groups (*P* = 0.72). The ADG (1.09 kg ± 0.13) of LF was lower (*P* = 0.01) than that of HF (1.63 kg ± 0.20). The expressions of 10 immune-related genes were upregulated (FC ≥ 1.2; *P* ≤ 0.05) in HF steers; these genes were involved in viral pathogen recognition and eradication, defense against intracellular and extracellular pathogens and parasites, and immune response homeostasis. A total number of 42 carbonyl-containing metabolites and 229 amine/phenol-containing metabolites were identified in the plasma samples of both groups. No alteration in carbonyl-metabolome was detected. Ten metabolites with immunomodulatory, anti-inflammatory, and reactive oxygen-scavenging properties were greater (FDR ≤ 0.05) in HF steers, whereas eight metabolites including arginine, phenylalanine, guanidoacetic acid, and aspartyl-threonine were greater in LF steers. This study demonstrated that beef steers with divergent ADG had altered plasma amine/phenol metabolome and immune-related gene expressions in the blood. Notably, plasma metabolites and immune-related genes of great health benefits were greater in steers with high ADG.

## INTRODUCTION

Feed efficiency continues to be of significant interest due to rising feed costs (Connor, 2015). Thus, several studies have evaluated several ways to optimize feed efficiency (Artegoitia et al., 2017; Thallman et al., 2018) via increased feed nutrient use for better growth performance (Parsons et al., 2012). Metabolism of macronutrients and energy yield from feeds have been shown to be associated with growth performance of animals (Herd et al., 2004). Thus, growth rate of animals depends, in part, on the supply of amino acids and energy-yielding substrates delivered to the tissues (Poppi and McLennan, 1995). Concentrations of blood metabolites are often used to assess the health and nutritional status of animals because these blood metabolites are common intermediate products of nutrient metabolism (Ndlovu et al., 2007).

In recent years, advent of metabolomics technologies has provided an opportunity to comprehensively analyze multiple metabolites in biofluids such as blood (Zhang et al., 2015). Chemical isotope labeling (CIL) liquid chromatography mass spectrometry (LC–MS) is a powerful metabolomics technique to analyze metabolites based on chemical groups (Zhao et al., 2019). This technique provides comprehensive analysis, with high accuracy, of metabolites containing amine/phenol group, which are common intermediate products of amino acid metabolism, and carbonyl group, which are common intermediate products of energy metabolism (Zhao et al., 2019). In this study, we hypothesized that beef steers with divergent average daily gain (ADG) would have different concentrations of plasma metabolites associated with amino acid and energy metabolism.

Due to the functional role of nutrient metabolisms (particularly amino acids) on animal health and immunity, the contribution of innate and adaptive immune competence to ADG divergence cannot be ignored. It is believed that animals that possess a better ability to effectively contend with pathogens and inflammatory stress will be more feed efficient. For example, a study by Foote et al. (2017) reported that beef steers with greater ADG adjusted for similar DM intake have a greater capacity to handle foreign substances that are extrinsic to the normal body metabolism. Thus, our second hypothesis was that beef steers with divergent ADG would have altered expressions of whole-blood immune-related gene expressions. The objective of this study was to determine plasma concentrations of amine/phenol and carbonyl-containing metabolites and whole-blood immune-related gene expressions in beef steers with divergent ADG.

## MATERIALS AND METHODS

The research procedures were approved by the Institutional Animal Care and Use Committees of Kentucky State University (protocol number 19-001).

### Animals, Housing, and Feeding

Forty-eight (48) recently weaned Angus crossbred beef steers (21 d postweaning; 210 ± 8.5 kg of body weight) from a single source were housed in individual slatted floor pens (2.44 × 14.63 m^2^). The steers were individually fed ad libitum a corn silage-based total mixed ration containing 79% corn silage and 21% grain mix containing distillers grain, soybean meal, and limestone (CP = 14.5% and NE_g_ = 1.10 Mcal/kg; Table 1) with free access to water. The feeding period was 42 days after a 21-day adaptation to the corn silage-based diet (63 days total). The steers had no growth-promoting implants and were not fed ionophores.

**Table 1.** Ingredient and chemical composition of the diet^1^

Ingredient, %DM	% of dietary DM
Corn silage	79.7
Dehydrated distillers grain	9.06
Soybean meal	9.28
Limestone	0.42
Deccox^2^	0.03
Vitamin and mineral premix^3^	1.51
Nutrient analysis^4^	
DM, %	44.5
CP, %	14.7
aNDF, %	38.6
ADF, %	21.5
EE, %	3.50
Ca, %	0.87
P, %	0.63
TDN, %	72.6
NE_m_, Mcal/kg	1.72
NE_g_, Mcal/kg	1.10

^1^Chemical composition of complete diets calculated from analysis and concentration of individual ingredients.

^2^Contains 6% decoquinate for the prevention of coccidiosis (Zoetis Inc.).

^3^Guaranteed analysis: 15% Ca; 7.5% P; 20% salt; 1% Mg; 1% K; 3,600 mg/kg Mn; 12 mg/kg Co; 1,200 mg/kg Cu; 3,600 mg/kg Zn; 27 mg/kg Se; 60 mg/kg I; 660,000 IU/kg vitamin A; 660 IU/kg vitamin E; and 66,000 IU/kg vitamin D.

^4^DM = dry matter; CP = crude protein; aNDF = neutral detergent fiber (amylase treated); ADF = acid detergent fiber; EE = ether extract; TDN = total digestible nutrients; NE_m_ = net energy of maintenance; NE_g_ = net energy of gain.

### Dry Matter Intake and Body Weight Measurement

The quantity of feed offered to and refused (as-fed) by each steer was recorded daily. Dry matter content of diet refused and offered was obtained by drying daily samples of the diets in a forced-air oven at 56 °C for 48 h. Daily DM intake was determined by subtracting the daily DM refused from the daily DM. Body weights of the steers were obtained before morning feeding, after about 10 h of feed withdrawal on days 0 and 42. Total weight gain was determined by subtracting the initial weight on day 0 (after the 21 d adaptation period) from the final weight on day 42. ADG was then determined by dividing the total weight gain by the number of experimental days (42 d). Steers with the lowest (LF: *n* = 8) and highest ADG (HF: *n* = 8) were selected from the 48 steers.

### Blood Sample Collection

Approximately 10 mL of blood was taken from all steers before the morning feeding on day 42 from the coccygeal vessels into tubes containing sodium heparin (Vacutainer, Becton Dickinson, Franklin Lakes, NJ). The tubes containing the blood were placed on ice immediately after collection. Immediately after collection, a subsample (500 µL each) of the whole blood was transferred into RNA-protect tubes (cat. no. 76554; Qiagen, Valencia). These RNA-protect tubes contain a reagent that lyses blood cells and stabilizes intracellular RNA. These were thereafter stored at −20 °C until RNA extraction and mRNA expression analysis of innate and adaptive immune-related genes were done. Another subsamples were used for plasma preparation within 30 min of collection by centrifugation at 2,500 × *g* for 20 min at 4 °C, and thereafter stored at −20 °C until untargeted metabolomics analysis using CIL/LC–MS was done.

### RNA Extraction, Complementary DNA (cDNA) Preparation, and Immune Gene Expression Analysis

Whole blood samples from LF (*n* = 8) and HF steers (*n* = 8) were analyzed for expression of immune-related genes. Total RNA was extracted using RNeasy Protect Animal Blood kit (cat. no. 73224; Qiagen) following the manufacturer’s recommended protocol. Synthesis of cDNA was done using the RT^2^ First Strand Kit (cat. no. 330401; Qiagen) following the manufacturer’s instructions. Expression of 84 genes related to innate and adaptive immune responses was analyzed using the RT^2^ Profiler cow innate and adaptive immune responses PCR Array (PABT-052ZA; Qiagen) according to the manufacturer’s instructions. Details of the procedure have been previously reported (Adeyemi et al., 2019). The PCR array contained 84 adaptive and innate immune-related genes, five housekeeping genes (β-actin, glyceraldehyde-3-phosphate dehydrogenase, hypoxanthine phosphoribosyltransferase 1, TATA box-binding protein, and tyrosine 3-monooxygenase), one genomic DNA control, three reverse transcription controls, and three positive PCR controls. Real-time PCR was done using a QuantStudio 7 Flex Real-Time PCR System (Applied Biosystems, Foster City, CA) with the following cycling conditions: 95 °C for 10 min, 40 cycles of denaturation at 95 °C for 15 s and 60 °C for 1 min. The mRNA expression levels of the 84 genes (delta cycle threshold (Ct)-values) were calculated by the difference between the average Ct value of the housekeeping genes and the Ct value of each of the immune genes.

### CIL-LC–MS-Based Metabolomics

In-depth untargeted metabolome profile of the plasma samples collected from the LF (*n* = 8) and HF steers (*n* = 8) was done using CIL/LC–MS-based technique. This technique uses a differential 12C- and 13C-dansylhydrazine labeling to change the chemical and physical properties of metabolites to enable efficient separation with LC and ionization by electrospray ionization MS (Zhao et al., 2017). In this study, metabolites containing the amine/phenol (amine/phenol-metabolome) and carbonyl groups (carbonyl-metabolome) were analyzed. One of the samples was damaged during carbonyl-metabolome analysis. As a result, eight HF and seven LF samples were analyzed for carbonyl-containing metabolites. Sample amount normalization was done using liquid chromatography–ultraviolet quantification of the dansyl-labeled metabolites (Wu and Li, 2012) and relative quantification of the metabolites based on peak ratio values was performed on an Agilent 1100 LC system (Agilent Technologies Inc., Palo Alto, CA) connected to a Bruker Impact HD quadrupole time-of-flight MS (Bruker Daltonics Inc., Billercia, MA). Details of sample preparation and methods used have been previously reported (Mung and Li, 2017).

#### **Metabolite data processing**.

Processing of raw LC–MS data was performed using IsoMS Pro 1.0 according to previously described procedures (Mung and Li, 2017). Peak pairs whose mean (sample)/mean (blank) was ≤4.0 and/or with no data present in at least 80% of the samples were removed. IsoMS-Quant was used to generate the final metabolite-intensity table (Huan and Li, 2015).

#### Metabolite identification.

Identification of peak pairs was done using a labeled metabolite library (CIL Library; amine/phenol and carbonyl (ketone and aldehyde) channel) based on accurate mass and retention time (Huan and Li, 2015). This label standard library contains 1060 unique human endogenous metabolites including 711 amines/phenols and 77 carbonyls (Zhao et al., 2019). Linked identity library was used to identify some of the peak pairs that could not be identified using CIL library based on accurate mass and predicted retention time matches (Li et al., 2013). Linked identity library contains metabolic-pathway-related metabolites extracted from the KEGG database (Zhao et al., 2019).

### Statistical Analysis

Growth performance variables such as ADG, initial and final body weights, and DM intake of LF (*n* = 8) and HF steers (*n* = 8) were analyzed using the GLIMMIX procedure of SAS version 9.4 (SAS Institute Inc., Cary, NC), with treatment included as a fixed effect. Significant effects were declared at *P* ≤ 0.05. Values of initial weight of the steers were included as a covariate for the final body weight.

Analysis of the immune-related gene expression data was performed with the GeneGlobe Data Analysis Center (https://geneglobe.qiagen.com/us/analyze/) using the delta-delta-Ct (ΔΔCt) method [(CT_gene of interest_ – CT_housekeeping genes_)_HF_ – (CT_gene of interest_ – CT_housekeeping genes_)_LF_] with normalization of the raw data using the arithmetic mean of five housekeeping genes. Immune genes with FC ≥ 1.2 or ≤ 0.83 having *P*-value ≤ 0.05 were considered to be differentially upregulated or downregulated relative to LF, respectively.

The metabolite intensity tables for the amine/phenol and carbonyl metabolome were imported separately into Metaboanalyst 4.0 software for statistical analysis. The data were first log-transformed and auto-scaled prior to statistical testing. Metabolites that differed (false discovery rate (FDR) ≤ 0.05) between LF and HF were first identified using *t*-test with a volcano plot. If there were metabolites that were significantly different based on FDR ≤ 0.05, partial least squares discriminant analysis (PLS-DA) scores plot was generated to visualize the differences and to identify the metabolites that were powerful discriminators between the two groups, based on their variable importance in projection (VIP) values. In this study, metabolites with VIP values ≥ 2 were considered as the powerful group discriminators. Cross validation was done to evaluate the fit and prediction power of the PLS-DA model. The utility of metabolites with VIP ≥ 2 to classify the ADG groups was further tested using a receiver operating characteristic (ROC) curves as calculated by the ROCCET web server (Xia et al., 2013). Area under the curve (AUC) from ROC curve, a value that combines sensitivity and specificity for a diagnostic test was used. Metabolites having AUC > 0.90 were considered excellent classifiers of ADG (Xia et al., 2013).

## RESULTS

The results of the growth performance of LF and HF steers are shown in Table 2. The initial body weight and average daily DM intake of the steers were similar between the two groups (*P* > 0.05). The final body weight was greater (*P* = 0.01) for HF compared with LF steers; consequently, the ADG of LF (1.09 kg/day) was lower (*P* = 0.01) than that of HF (1.63 kg/day).

**Table 2.** Growth performance of the beef steers with divergent average daily gain

	LF, *n* = 8	HF, *n* = 8	SEM	*P*-value
Average daily gain, kg/day	1.09^b^	1.63^a^	0.07	0.01
Initial body weight, kg	229	225	5.21	0.73
Final body weight, kg	274^b^	293^a^	2.89	0.01
Dry matter intake, kg/day	6.08	6.04	0.23	0.92

LF = beef steers with lowest average daily gain; HF = beef steers with highest average daily gain; *n* = number of animals in each group; SEM = standard error of mean.

^a,b^Within a row, treatment means with different superscripts differ (*P* ≤ 0.05).

### Immune-Related Gene Expression Associated with Divergent ADG

A total of 11 genes were differentially expressed between HF and LF steers. The mRNA expressions of 10 immune genes (IRF3, TLR3, CCR4, MAPK3, TYK2, STAT3, STAT4, STAT6, CCR8, and GATA3) were upregulated in HF steers, whereas a pro-inflammatory cytokine, IL-2, was upregulated in LF steers (Table 3).

**Table 3.** List of differentially expressed whole-blood immune genes between LF and HF steers

Gene symbol	Gene name	Fold change^1^	*P*-value
IRF3	Interferon regulatory factor 3	1.35	<0.01
TLR3	Toll-like receptor 3	1.40	0.01
CCR4	Chemokine (C–C motif) receptor 4	1.49	0.01
MAPK3	Mitogen-activated protein kinase 3	1.19	0.01
TYK2	Tyrosine kinase 2	1.30	0.03
STAT3	Signal transducer and activator of transcription 3	1.23	0.04
STAT4	Signal transducer and activator of transcription 4	1.35	0.02
STAT6	Signal transducer and activator of transcription 6	1.20	0.05
CCR8	Chemokine (C–C motif) receptor 8	1.90	0.03
GATA3	GATA binding protein 3	1.31	0.05
IL2	Interleukin 2	0.51	0.02

LF = beef steers with lowest average daily gain; HF = beef steers with highest average daily gain.

^1^Fold change (relative to control) = 2^−ΔΔCt^ = [(CTgene of interest – CTreference genes)_HF_ – (CTgene of interest – CTreference genes)_LF_]. Only genes with both fold change ≥1.2 or ≤0.83, relative to LF, and *P* ≤ 0.05 are shown.

### Amine/Phenol-Metabolome Associated with Divergent ADG

**Figure 1.**
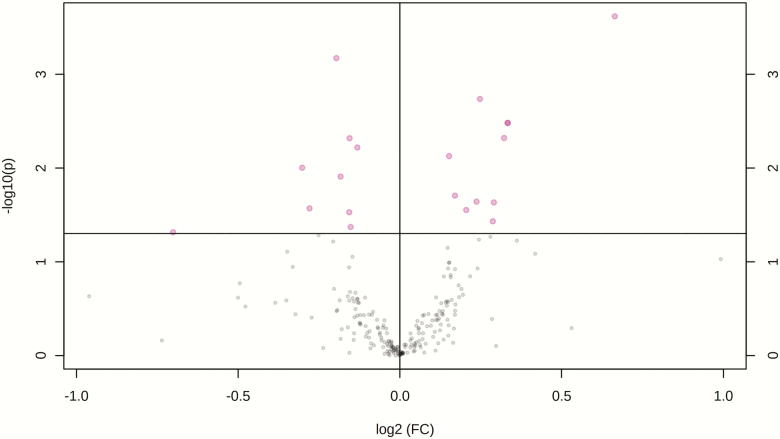
Volcano plot showing the differential amine/phenol-containing plasma metabolites (FDR ≤ 0.05) between LF and HF steers. LF = beef steers with lowest average daily gain; HF = beef steers with highest average daily gain. Purple dots represent the differential metabolites.

**Figure 2.**
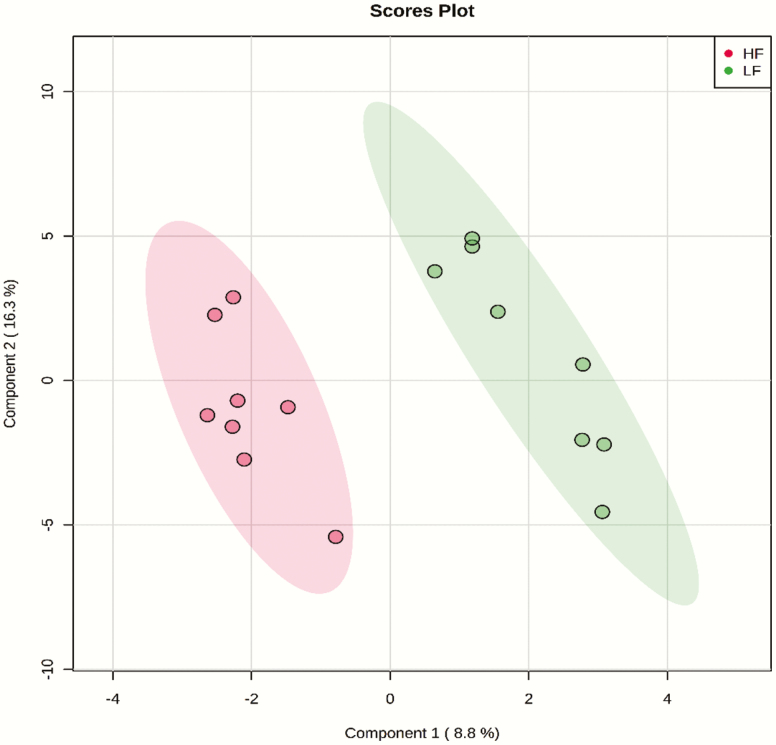
PLS-DA scores plot of amine/phenol-metabolome of LF and HF steers. LF = beef steers with lowest average daily gain; HF = beef steers with highest average daily gain.

A total number of 229 amine/phenol-containing metabolites were identified in the plasma samples of LF and HF steers (Table S1). The volcano plot analysis revealed 18 differential (FDR < 0.05) metabolites between LF and HF steers (Figure 1); 10 metabolites including prolyl-valine, prolyl-isoleucine, and prolyl-leucine were greater in HF steers while eight metabolites including phenylalanine and arginine were greater in LF steers. Multivariate analysis (PLS-DA) showed clear separation between the plasma amine/phenol metabolome between the two groups of steers (Figure 2). Cross-validation analysis confirmed the validity of the PLS-DA model (Supplementary Figure S1).

**Figure 3.**
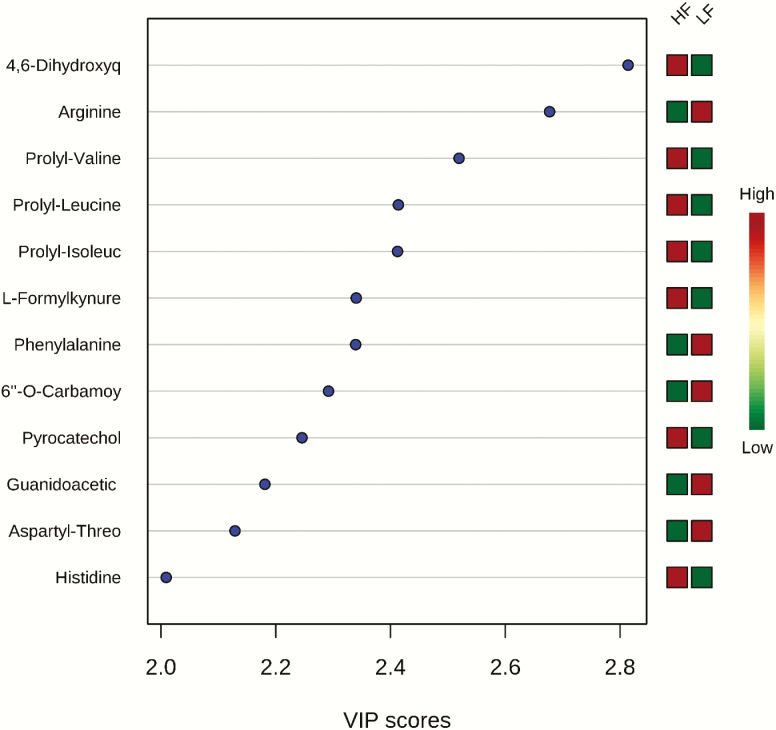
Differential metabolites between LF and HF steers ranked by variable importance in projection (VIP) values. LF = beef steers with lowest average daily gain; HF = beef steers with highest average daily gain. Only differential metabolites with VIP value ≥ 2.0 are shown.

**Figure 4.**
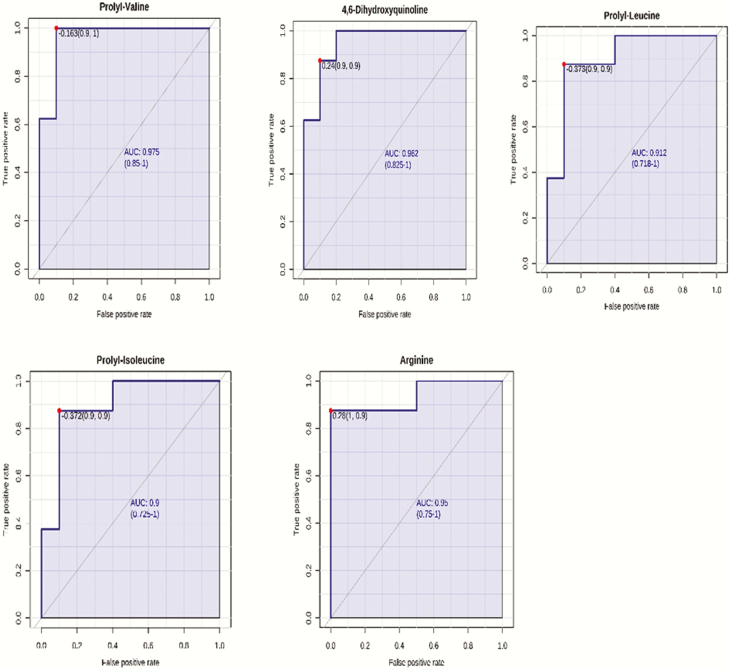
Receiver–operator characteristic curves of prolyl-valine, 4,6-dihydroxyquinoline, prolyl-leucine, prolyl-isoleucine, and arginine.

**Figure 5.**
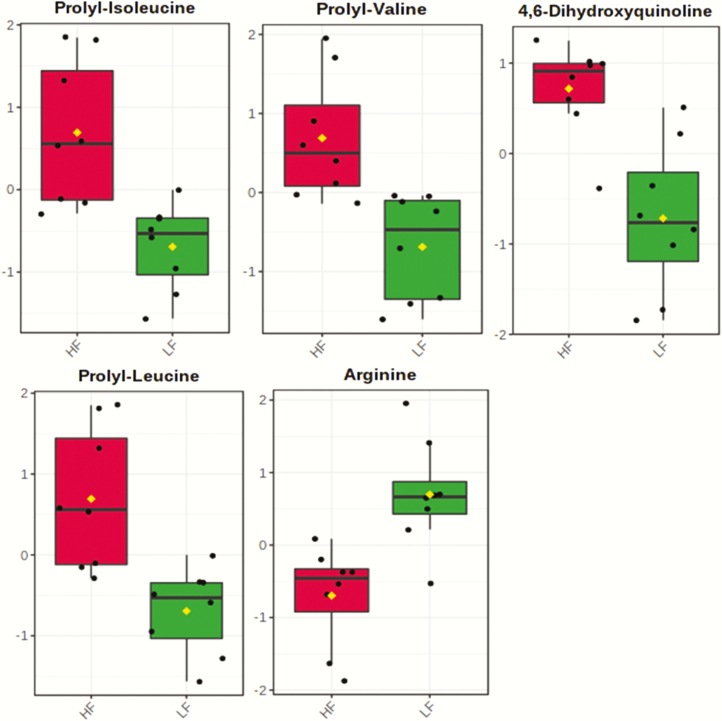
Box plot showing the distribution of prolyl-valine, prolyl-isoleucine, prolyl-leucine, 4,6-Dihydroxyquinoline, and arginine between LF and HF steers. LF = beef steers with lowest average daily gain; HF = beef steers with highest average daily gain.

The ranking of the metabolites by VIP values ≥ 2 showed that 12 metabolites contributed the most to the separation of the two groups of steers. 4,6-Dihydroxyquinoline, prolyl-valine, prolyl-leucine, prolyl-isoleucine, l-formylkynurenine, pyrocatechol, and histidine were greater in HF steers, whereas arginine, phenylalanine, 6″-*O*-carbamoylkanamycin A, guanidoacetic acid, and aspartyl-threonine were greater in LF steers (Figure 3). The utility of the top 5 metabolites with the highest VIP values to classify divergent ADG was tested using an ROC analysis. The results of the ROC analysis revealed that these five metabolites (prolyl-valine, 4,6-dihydroxyquinoline, arginine, prolyl-isoleucine, and prolyl-leucine) with respective AUC of 0.96, 0.95, 0.94, 0.90, and 0.90 had sufficient specificity and sensibility to classify divergent ADG (Figure 4). The box plots showing the distributions in LF and HF steers are shown in Figure 5.

### Carbonyl-Metabolome Associated with Divergent ADG

**Figure 6.**
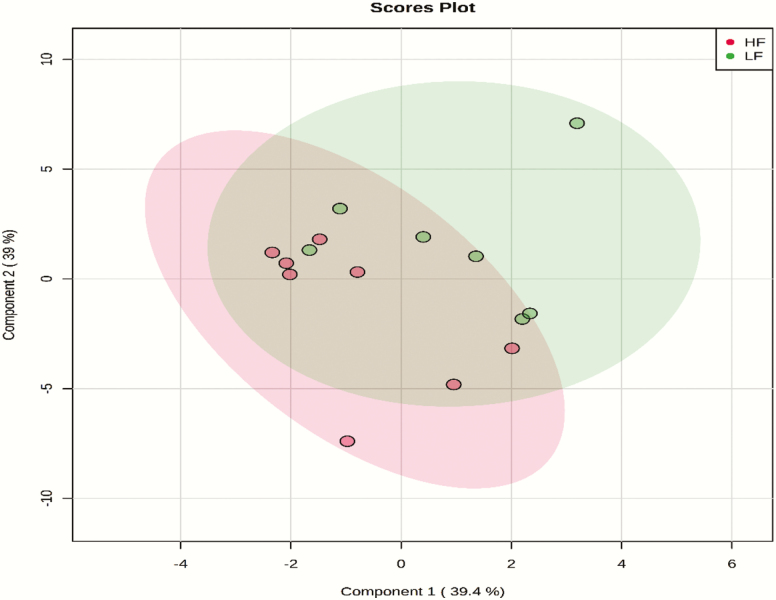
PLS-DA scores plot of carbonyl-metabolome of LF and HF steers. LF = beef steers with lowest average daily gain; HF = beef steers with highest average daily gain.

A total number of 42 carbonyl-containing metabolites were identified in the plasma samples of LF and HF steers (Supplementary Table S2). The volcano plot analysis showed that no metabolites were different (FDR > 0.05) between the two groups of steers (Supplementary Figure S2). This was confirmed by PLS-DA scores plot which revealed an overlap between the plasma carbonyl-metabolome of the two groups of steers (Figure 6). These results were further confirmed by the results of ROC analysis which showed that all the carbonyl-containing metabolites had AUC < 0.80 (data not shown).

## DISCUSSION

The primary limitation of this study was that these results were based on blood samples collected at one time point because blood metabolome is dynamic and varies in response to exogenous metabolites, such as those supplied via diet, and changes in gene expression (Smith et al., 2020). Therefore, as presented in this study, comparison of blood metabolome profiles among animals at a particular time point should be done using fasting plasma sample which is more reflective of modulations in endogenous metabolism (Smith et al., 2020), and should be accompanied by gene expression analysis of interest, such as immune gene expression profile. It is important to note that this is not a validation study, but rather a study to initially identify potential mechanisms that cause divergence in ADG of beef cattle which may be used to inform future studies aimed at improving beef cattle productivity. Another possible concern about this study was the use of ADG as the feed efficiency-related trait because residual feed intake has been widely used for classifying feed efficiency in beef cattle (Koch et al., 1963). However, ADG with similar DM intake is reported to provide the most accurate mathematical description of cause and effect and is recommended as the preferred measure of biological efficiency (Koch et al., 1963; Thallman et al., 2018).

### Expressions of Immune-Related Genes Associated with Divergent ADG

Compared with LF, there was an increase (FC ≥ 1.2, *P* ≤ 0.05) in the mRNA expressions of TLR-3 and IRF-3 in HF steers. Toll-like receptors are present in immune cells, including dendritic cells and macrophages and play a key role of initiating an innate immune response against invading microbial pathogens (Billack, 2006). Toll-like receptors are able to recognize specific molecular patterns from invading pathogens (and nonpathogens) in order to effectively initiate an appropriate host defense by inducing interferon α/β (Janssens and Beyaert, 2003) that are known to interfere with virus replication and impede their spread (Stetson and Medzhitov, 2006). TLR-3 mediates viral double-strand RNA recognition (Vercammen et al., 2008), and IRF-3 is essentially involved in regulating TLR-3-induced interferon-β gene transcription in response to viral infections (Kawai and Akira, 2007). Increased mRNA expressions of TLR-3 and IRF-3 genes in HF steers suggest that they had a better ability than LF steers to quickly recognize viral pathogens and initiate an appropriate innate antiviral defenses.

There were greater (FC ≥ 1.2, *P* ≤ 0.05) mRNA expressions of GATA-3, CCR4, and CCR8 genes in HF steers, compared with LF steers. These genes play an important role in differentiation and recruitment of T-helper 2 cells (Th2). GATA-3 is the key regulator of Th2 differentiation (Zheng and Flavell, 1997). Both CCR4 and CCR8 are expressed on T cells and are essential for recruiting Th2 cells from the blood to the skin (D’Ambrosio et al., 1998). T-helper 2 cells are group of T cells that protect against external parasites, such as helminth parasites (Pearce et al., 1991). Increased expressions of these genes suggest that HF steers likely have a greater ability to effectively fight off parasitic infections than LF steers.

Increased mRNA expressions of tyrosine kinase, MAP kinase, STAT3, STAT4, and STAT6 were observed in the whole blood of HF steers. Signal transducers and activators of transcription are transcription factors that can be activated by several signaling proteins such as tyrosine and MAP kinases, interferons, growth factors, and interleukins (Tkash et al., 2013), and are essential for regulating cell differentiation and growth (Levy and Darnell, 2002). STAT3 is required for inducing retinoic acid receptor-related orphan receptor γ, a specific transcription factor for Th17 cells, which participate in extracellular bacterial and fungal eradication (Cipollini et al., 2019). STAT4 polarizes Th1 cells which are involved in intracellular pathogen elimination (Luckheeram et al., 2012). STAT6 activation inhibits Th1 polarization and is essential for Th2 signaling (Seif et al., 2017). Th1 cells elicit cell-mediated immunity by producing proinflammatory cytokines while Th2 cells evokes humoral-mediated immunity by producing anti-inflammatory cytokines which counteract the Th1-mediated response (Torre et al., 2002); thus, the balance between Th1- and Th2-mediated responses is needed for a healthy and homeostastic immune system. Increased expressions of these STAT proteins in HF steers suggest a balance between Th1 and Th2 activities, which is essential for a healthy and homeostastic immune system.

Expression of an inflammatory cytokine, IL-2, was downregulated (FC ≤ 0.83, *P* ≤ 0.05) in HF steers. Interleukin-2 is one of the several pro-inflammatory cytokines that are produced by Th1 cells (Viallard et al., 1999). It is well known that excessive concentrations of pro-inflammatory cytokines is an indication of inflammatory stress response and can lead to or worsen inflammatory reactions (Zhang and An, 2007). Several studies have shown that increased concentrations of pro-inflammatory cytokines are associated with decreased growth rate and efficiency of feed utilization in animals (Evock-Clover et al., 1997; Steiger et al., 1999) because nutrients that are meant to be used for tissue growth are redirected toward fueling the immune system to fight off the inflammation (Mani et al., 2012). Thus, it is reasonable to speculate that less nutrients are directed toward anabolic process in LF, compared with HF steers, which partially explains their lower ADG. Taken together, since animals are constantly being exposed to several stressors such as physical and microbial factors (viruses, live bacteria, and dead bacteria) that may predispose animals to inflammatory stresses (Holck et al., 1998; Smith, 1998); increased expressions of beneficial immune-related genes such as STAT proteins, MAP kinase, TLR-3, and IRF-3 which are involved in protection against inflammatory reactions and decreased expression of pro-inflammatory cytokine, IL-2, suggest that HF steers are better than LF steers, at fighting off infections without causing inflammatory reactions.

### Amine/Phenol- and Carbonyl-Metabolome Associated with Divergent ADG

Levels of DM intake have been shown to alter levels of nutrients, particularly amino acids and energy metabolites, in the blood circulation (Wang et al., 2019). In this study, DM intake did not differ between the two groups of steers, which suggests that alteration in plasma metabolome observed in this study was not due to differences in DM intake. In the present study, plasma amine/phenol-metabolome was different between steers with low and high ADG. The plasma concentrations of peptides containing proline and branched chain amino acids (BCAA) (prolyl-valine, prolyl-leucine, and prolyl-isoleucine) were greater in HF steers, whereas free amino acids such as arginine and phenylalanine were reduced. This may be due to differences in intestinal peptide uptake and/or tissue protein turnover. Protein turnover and tissue metabolism have been reported to account for up to 37% of variation in feed efficiency in beef cattle (Richardson and Herd, 2004). Elolimy et al., 2019 reported an association between residual feed intake and protein turnover and nutrient transporters in ruminal epithelium of beef cattle. In their study, there was a reduced abundance of intracellular enzymes associated with protein degradation and reduced abundance of amino acid transporters in the ruminal epithelium of highly efficient cattle. In another study, a decrease in muscular protein degradation was observed in most-efficient beef steers compared with less-efficient ones (Blank et al., 2017). Thus, it is reasonable to speculate that increased concentrations of dipeptides in plasma of HF steers may be a function of greater abundance or activity of peptide transporter (PepT1) in the gut (small intestine, rumen, and omasum) and/or differences in intracellular protein turnover (such as reduced activity or abundance of cytoplasmic peptidase), leading to increased flow of intact peptides into the blood. In addition to peptide transport via PepT1, peptides can be absorbed through paracellular movement and via cell-penetrating peptides which can penetrate the plasma membrane (Borrelli et al., 2018). It has been demonstrated that transport of peptides via PepT1 into the cell is more energy-efficient than transport of a free amino acid (Daniel, 2004). In fact, an amino acid is absorbed at a faster rate in the intestine if it is a component of a dipeptide or a tripeptide than as a free amino acid. Therefore, greater absorption of BCAA in form of peptides in the gut may minimize energy expenditure as well as increase amino acid availability for tissue protein synthesis, which probably explains the improved ADG of HF steers.

In addition to the nutritional value of blood peptides as a readily available source of amino acids for tissue protein synthesis, BCAA play a key role in up-regulating innate and adaptive immune responses (Zhang et al., 2017). Moreover, di- and tripeptides peptides, including those containing proline, have antimicrobial, antioxidant, and immunomodulatory activities (Li-Chan, 2015). These suggest that HF steers are in a better health and immune status, which explains the immune-related gene expression results. The fact that plasma concentrations of aspartyl-threonine, phenylalanine, and arginine were lower in HF steers may be because the absorption of BCAA-based peptides was favored at the expense of these amino acids. Another explanation for reduced concentration of arginine in HF steers may be that it was metabolized to proline to make up for a possible reduced concentration of free proline in the blood because all of the dipeptides that were greater in HF steers had proline as one of their components.

Increased concentrations of 4,6-dihydroxyquinoline and l-formylkynurenine, both metabolites from tryptophan metabolism via the kynurenine pathway (Moffett and Namboodiri, 2003), are an evidence of improved health and wellbeing of the HF steers. Enzymes such as indoleamine 2,3-dioxygenase that converts  l-tryptophan to l-formylkynurenine, and kynurenine 3-monooxygenase that converts 5-hydroxykynurenamine to 4,6-dihydroxyquinoline, play a significant role in immune homeostasis. In addition, kynurenine molecules can scavenge hydrogen peroxides and superoxides. In fact, kynurenine metabolites in the blood can inhibit reactive oxygen species (ROS) production by activated neutrophils (Genestet et al., 2014). Reactive oxygen metabolites (hydrogen peroxide and superoxide) are products of normal metabolic processes and, when not adequately removed, impair the performance and health of animals via peroxidative damage to nutrients such as lipids and macromolecules (Miller et al., 1993). Increased concentrations of kynurenine molecules in the blood suggest that HF steers have a better ability than LF steers to effectively and safely remove ROS.

The blood concentrations of metabolites containing carbonyl group such as ketones and aldehydes reflect the energy status of animals as they are common intermediate products of energy metabolism (Zhao et al., 2017; Adeyemi et al., 2020). The lack of difference in plasma carbonyl-metabolome between HF and LF steers was unexpected but not surprising because one of the major energy substrates, glucose, is tightly regulated and is in continuous supply via gluconeogenesis (Aschenbach et al., 2010; Clemmons et al., 2017). No studies that evaluated plasma carbonyl-metabolome in beef steers with varying feed efficiency-related traits could be found. However, similar results have been reported in previous studies that assessed selected blood metabolites such as blood glucose and NEFA concentrations of animals divergent in residual feed intake, a common measure for assessing feed efficiency in beef cattle. For example, Clemmons et al. (2017) reported no differences in serum glucose and NEFA in Black Angus steers divergent in residual feed intake. In another study, plasma glucose concentration was similar in growing beef bull divergent in residual feed intake (Bourgon et al., 2017).

## CONCLUSION

This study demonstrated that beef steers with divergent ADG had differential expressions of immune-related genes of great health benefits in the blood. Notably, immune-related genes that are involved in viral pathogen recognition, defense against intracellular and extracellular pathogens and parasites, and immune response homeostasis were upregulated in HF steers. The altered immune-related gene expression profile was possibly explained by increased plasma concentrations of dipeptides containing BCAA residues (prolyl-valine, prolyl-leucine, and prolyl-isoleucine) and metabolites with anti-inflammatory and ROS-scavenging properties (4,6-dihydroxyquinoline and l-formylkynurenine) in HF steers. Future research is needed to determine the mechanisms that cause differences in the whole-blood immune-related gene expression and plasma concentrations of the aforementioned metabolites between the two groups and how these mechanisms can be employed to drive improved ADG and feed efficiency in beef steers.

## Supplementary Material

txaa074_suppl_Supplementary_Figure_S1

txaa074_suppl_Supplementary_Figure_S2

txaa074_suppl_Supplementary_Table_S1

txaa074_suppl_Supplementary_Table_S2
