# Supplementary figures and images for: Average daily gain divergence in beef steers is associated with altered plasma metabolome and whole blood immune-related gene expression
Source: Transl Anim Sci. 2020 May 27;4(3):txaa074. doi: 10.1093/tas/txaa074 (PMC7381838; doi:10.1093/tas/txaa074)

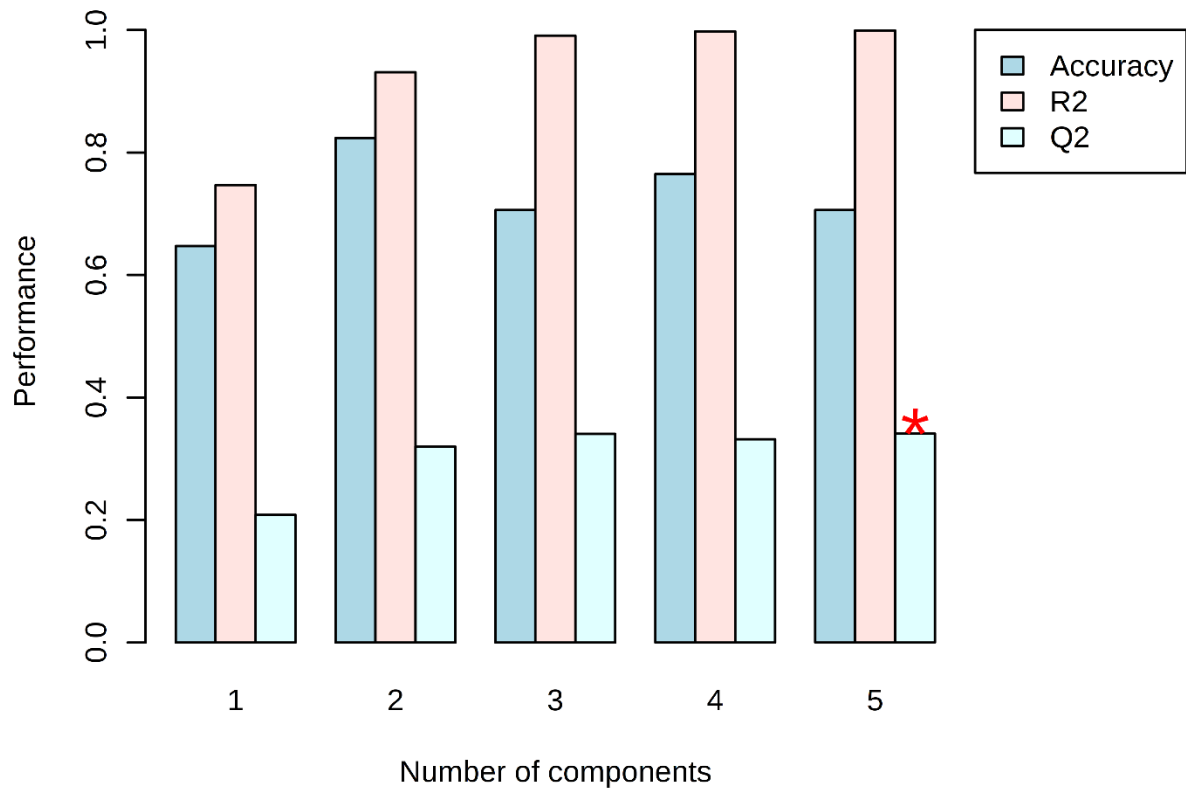

Supplementary figure 1. Cross validation results of the PLS-DA model

Supplement: txaa074_suppl_Supplementary_Figure_S1 [file txaa074_suppl_supplementary_figure_s1.pdf]
