# Supplementary material for: Average daily gain divergence in beef steers is associated with altered plasma metabolome and whole blood immune-related gene expression
Source: Transl Anim Sci. 2020 May 27;4(3):txaa074. doi: 10.1093/tas/txaa074 (PMC7381838; doi:10.1093/tas/txaa074)
Supplement: txaa074_suppl_Supplementary_Figure_S2 [file txaa074_suppl_supplementary_figure_s2.pdf]

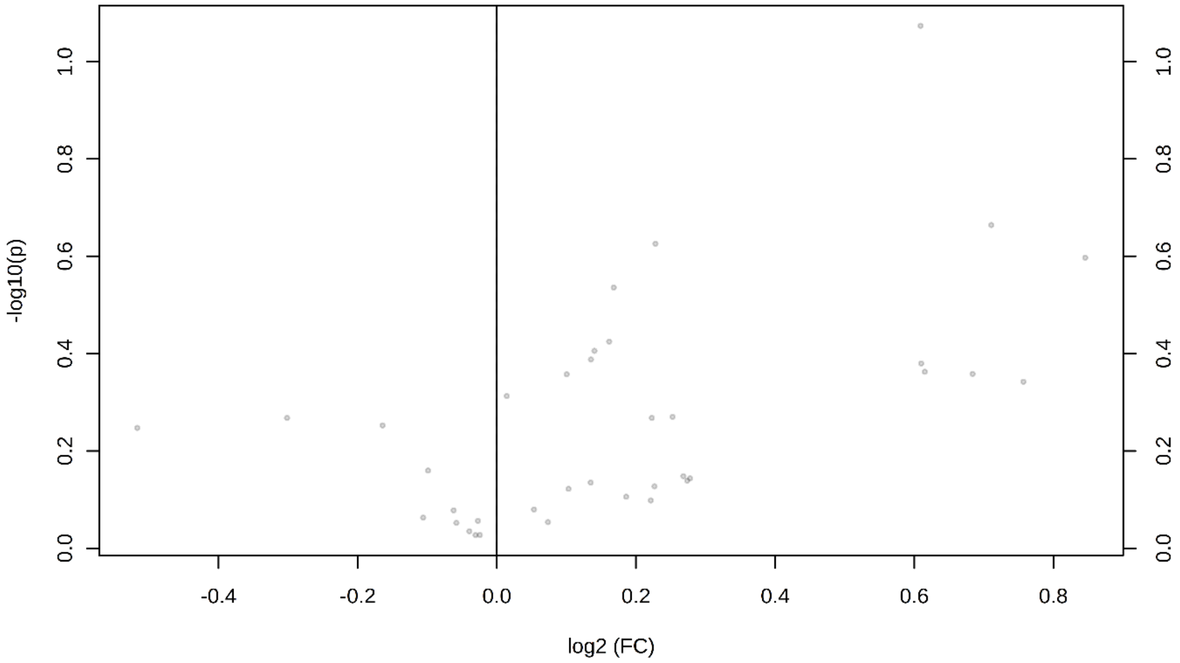

Supplementary Figure 2. Volcano plot showing changes in carbonyl metabolites between beef steers with divergent average daily gain
